# The Relationship Between Eosinophilic Esophagitis and Laryngeal Manifestations: Pathophysiology, Clinical Overlap, and Diagnostic Challenges

**DOI:** 10.3390/jcm15166451

**Published:** 2026-08-20

**Authors:** Ionut Costin, Mihai Dumitru, Ovidiu Berghi, Daniela Vrinceanu, Alina Lavinia Antoaneta Oancea, Andrei Cozea, Alexandru Moldoveanu, Georgeta-Carmen Fierbinteanu-Braticevici

**Affiliations:** 1 ENT Department, Carol Davila University of Medicine and Pharmacy, 020021 Bucharest, Romania; cristian.costin@stud.umfcd.ro (I.C.); vrinceanudana@yahoo.com (D.V.); 2 Allergology Department, Saint Mary Clinics and Laboratories, 061126 Bucharest, Romania; oberghi@yahoo.com; 3 ENT Department, Elias Clinical Hospital, 011461 Bucharest, Romania; dr.alina.oancea@gmail.com; 4 Internal Medicine and Gastroenterology Department, Carol Davila University of Medicine and Pharmacy, 020021 Bucharest, Romania; andrei.cozea@drd.umfcd.ro (A.C.); alexandru@moldoveanu.tel (A.M.); carmen.fierbinteanu@umfcd.ro (G.-C.F.-B.)

**Keywords:** eosinophilic esophagitis, laryngeal manifestations, laryngopharyngeal reflux, subglottic stenosis, chronic cough, aerodigestive disorders, eosinophilic inflammation

## Abstract

**Background:** Eosinophilic esophagitis (EoE) is a chronic type 2 immune-mediated esophageal disease defined by esophageal dysfunction and eosinophil-predominant inflammation. Beyond classic gastrointestinal symptoms, patients may present with aerodigestive complaints such as chronic cough, hoarseness, recurrent croup, stridor, vocal fold abnormalities, and subglottic stenosis, creating diagnostic overlap with laryngopharyngeal reflux, vocal fold dysfunction, asthma, allergic rhinitis, and other airway disorders. **Methods:** This scoping review mapped evidence on the relationship between EoE and laryngeal manifestations. PubMed/MEDLINE was searched using terms related to EoE, laryngeal disease, reflux, vocal fold pathology, and subglottic stenosis. Human English-language case reports, case series, observational studies, clinical trials, and reviews were narratively charted by study design, population, diagnostic criteria, laryngeal findings, reflux evaluation, treatment, and outcomes. **Results:** Laryngeal involvement in EoE appears clinically relevant but remains incompletely characterized. Direct eosinophilic infiltration of laryngeal or hypopharyngeal tissue is rare, whereas indirect mechanisms—including shared type 2 inflammation, epithelial barrier dysfunction, eosinophil and mast-cell mediators, reflux or microaspiration, and comorbid atopic airway disease—may contribute to symptoms and lesions. Pediatric patients may be especially likely to show airway-predominant presentations, including recurrent croup, chronic cough, and subglottic stenosis. Evidence is limited by heterogeneous definitions, inconsistent laryngoscopy or biopsy, variable reflux testing, and small observational reports. **Conclusions:** EoE should be considered in persistent or recurrent laryngeal symptoms, particularly when refractory to reflux or airway-directed therapy or associated with atopy. Multidisciplinary evaluation may improve recognition and management. Future studies should use standardized laryngeal assessment, pediatric case–control designs, and biomarker approaches linking EoE activity with objective laryngeal findings.

## 1. Introduction

Eosinophilic esophagitis (EoE) is a chronic, type 2 immune/antigen-mediated inflammatory disease of the esophagus, characterized by esophageal dysfunction and dense eosinophilic infiltration of the esophageal mucosa [1]. Normally the esophagus is devoid of eosinophils, so the presence of eosinophil infiltration denotes pathology. Over the past three decades, starting in the early 1990s, EoE has transitioned from a rare diagnosis in case reports to a leading cause of dysphagia and food impaction in both children and adults, with a prevalence estimated at 34–100 per 100,000 in Western countries making it one of the most actively studied gastrointestinal inflammatory diseases of modern times [2]. EoE represents a unique clinical entity bridging allergology and gastroenterology. Although it shares common type 2 inflammatory mechanisms with asthma, atopic dermatitis, allergic rhinitis, and chronic rhinosinusitis with nasal polyps, it is distinguished by its esophageal-specific involvement, its risk of developing fibrostenotic complications in the absence of adequate disease control, and the predominant contribution of food antigens as key environmental drivers of inflammation [2,3].

While EoE is classically defined by esophageal symptoms and histologic findings, a growing body of literature highlights its potential for extraesophageal manifestations, particularly involving the larynx and upper airway. Laryngeal symptoms such as chronic cough, hoarseness, and even subglottic stenosis have been reported in EoE patients, raising important questions about the pathophysiological links between esophageal and laryngeal inflammation, the potential for direct causation of laryngeal manifestations, and the diagnostic challenges posed by overlapping clinical presentations with other laryngeal disorders such as laryngopharyngeal reflux (LPR) and vocal fold dysfunction (VCD) [4,5].

This report provides a comprehensive analysis of the relationship between EoE and laryngeal manifestations, exploring the underlying immune mechanisms, tissue remodeling processes, clinical overlap, diagnostic complexities, and current evidence for direct or indirect causation. Special attention is given to pediatric presentations, the role of reflux and microaspiration, and the perspectives of otolaryngology and laryngology in the multidisciplinary management of these patients.

This scoping review was designed to map the available evidence on the relationship between eosinophilic esophagitis (EoE) and laryngeal manifestations, including possible direct eosinophilic involvement of laryngeal tissue, indirect inflammatory effects, and reflux-mediated injury. Although EoE is classically defined by esophageal symptoms and eosinophil-predominant inflammation on biopsy, reports of chronic cough, hoarseness, laryngitis, vocal fold abnormalities, and subglottic stenosis suggest that upper-airway manifestations may be clinically relevant and under-recognized.

## 2. Materials and Methods

This review followed the methodological framework and was reported according to PRISMA-ScR guidance, Table A1. The protocol for this scoping review was registered in the Open Science Framework (OSF; identifier: 9g6kc). The primary question was: what evidence links EoE with laryngeal pathology, and what mechanisms, diagnostic approaches, treatments, and research gaps have been described? PubMed/MEDLINE was used as the primary database.

PubMed/MEDLINE was searched on 1 March 2026 and served as the primary formal bibliographic database. The PubMed strategy combined terms for EoE with terms for laryngeal and upper-airway disease. The full reproducible search string was: (“eosinophilic esophagitis” OR “eosinophilic oesophagitis” OR EoE) AND (larynx OR laryngeal OR laryngitis OR “laryngopharyngeal reflux” OR LPR OR subglottic OR “subglottic stenosis” OR “vocal fold” OR dysphonia OR hoarseness OR cough OR croup OR stridor OR aerodigestive). Human studies in English were prioritized, and no date restriction was applied. Pediatric sensitivity searches were performed using additional terms such as “child,” “children,” and “pediatric.” In addition to PubMed/MEDLINE, supplementary source identification was performed through reference-list screening of relevant articles and targeted searches for specific presentations such as recurrent croup, chronic cough, laryngopharyngeal reflux, vocal fold dysfunction, subglottic stenosis, laryngeal cleft, aerodigestive dysfunction, and eosinophilic disease involving the hypopharynx or larynx. This supplementary process identified 18 additional records, which were included in the PRISMA flow diagram and evidence charting when eligible. Moreover, selected references were added during revision for context, Table S1.

Eligible sources included human case reports, case series, observational studies, clinical trials, and reviews that reported laryngeal signs or symptoms in patients with confirmed EoE, or eosinophilic inflammation involving laryngeal or hypopharyngeal tissue. Both adult and pediatric populations were included. Studies were excluded if they were animal-only reports, conference abstracts without extractable data, or non-English publications, although the language restriction was recorded as a limitation.

**Figure 1:**
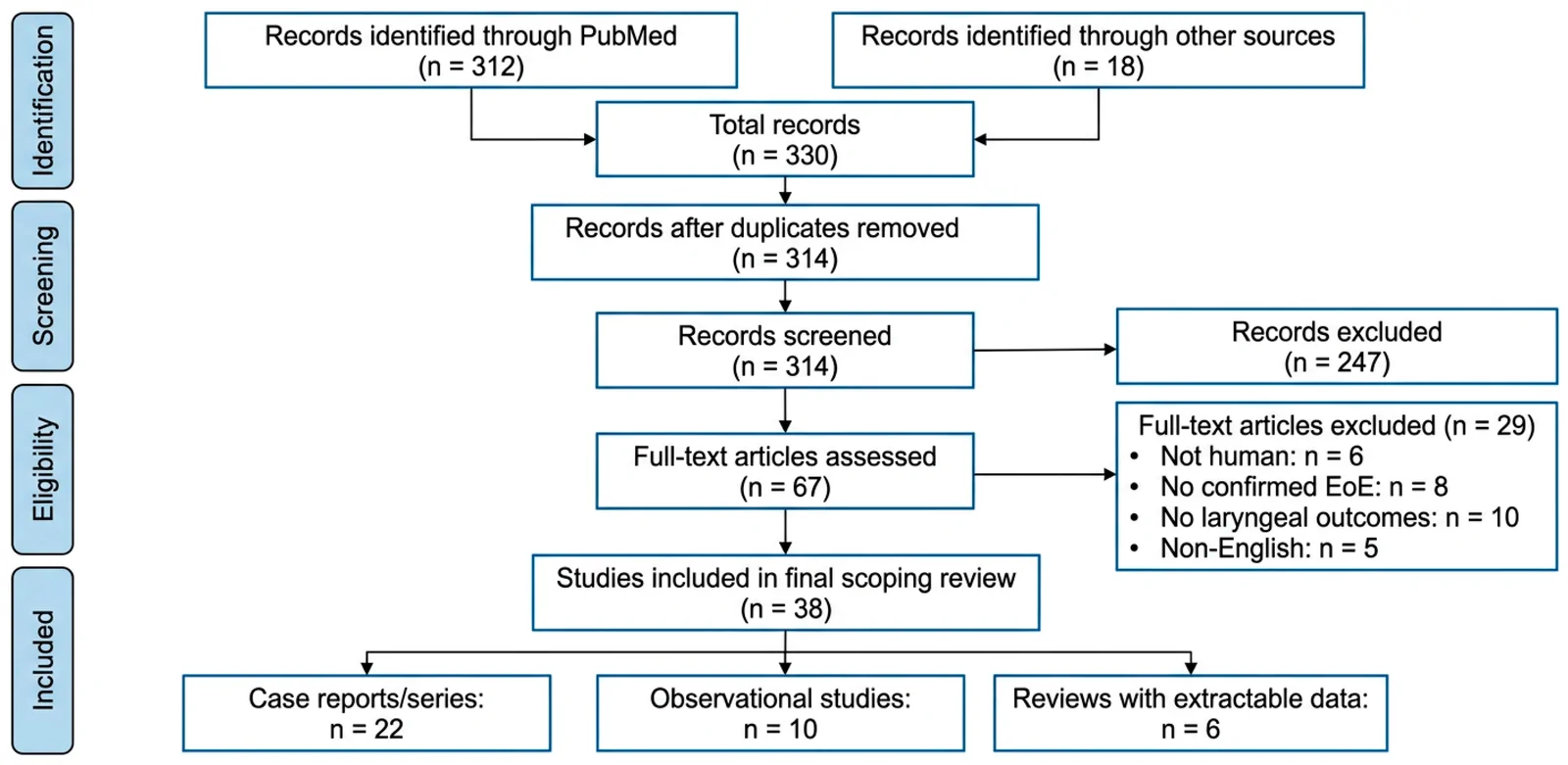
PRISMA diagram of the current scoping review.

All retrieved records were exported to reference-management software and deduplicated. Two reviewers independently screened titles and abstracts, followed by full-text review of potentially eligible records. Disagreements were resolved through discussion or consultation with a third reviewer. Study selection was documented using a PRISMA flow diagram, including the number of records identified, screened, excluded, and included, as shown in Figure 1.

Data were charted using a standardized extraction form. Extracted variables included citation details, country, study design, patient age group, atopic history, EoE diagnostic criteria, biopsy findings, laryngeal symptoms, laryngoscopy results, laryngeal or hypopharyngeal biopsy findings, reflux testing, treatments, and outcomes. Interventions of interest included proton-pump inhibitors, swallowed topical corticosteroids, dietary elimination therapy, biologic therapy, and surgical management when relevant.

Because scoping reviews aim primarily to map evidence rather than exclude studies on the basis of quality, formal critical appraisal was not required. However, when higher-level observational or interventional studies were identified, appropriate tools such as the Newcastle–Ottawa Scale or Cochrane risk-of-bias guidance were considered to support interpretation. Findings were synthesized narratively and organized into evidence tables describing study characteristics, laryngeal manifestations, diagnostic methods, proposed mechanisms, treatments, and outcomes.

As shown in Figure 1, the database and supplementary searches identified 330 records. After removal of duplicates, 314 records were screened by title and abstract, of which 247 were excluded. A total of 67 full-text articles were assessed for eligibility, and 38 sources were included in the final scoping synthesis. The included sources consisted mainly of case reports, small case series, retrospective observational studies, pediatric aerodigestive cohorts, and narrative or systematic reviews. Pediatric populations were prominently represented, particularly in studies addressing recurrent croup, chronic cough, subglottic stenosis, aerodigestive dysfunction, and airway evaluation by ENT or multidisciplinary aerodigestive teams. Adult evidence was more limited and consisted largely of isolated case reports, reviews, and interventional EoE studies in which laryngeal outcomes were not systematically assessed. Overall, the charted literature maps a heterogeneous evidence base in which direct eosinophilic involvement of laryngeal tissue is uncommon, while indirect mechanisms—including shared type 2 inflammation, reflux or microaspiration, and comorbid atopic airway disease—are more frequently discussed. Among the 38 included studies, 16 focused on pediatric populations, 6 were limited to adult populations, and 16 included mixed or combined age groups.

## 3. Results

### 3.1. Pathophysiology of EoE: Immune Mechanisms and Epithelial Responses

EoE is fundamentally a Th2-driven allergic disorder, triggered by food and environmental allergens in genetically predisposed individuals. Upon exposure to allergens, esophageal epithelial cells release “alarmin” cytokines such as thymic stromal lymphopoietin (TSLP), interleukin-33 (IL-33), and IL-25, which activate dendritic cells and group 2 innate lymphoid cells (ILC2s). These cells, in turn, secrete Th2-polarizing cytokines—IL-4, IL-5, and IL-13—amplifying the inflammatory cascade [6,7].

IL-5 is critical for eosinophil differentiation, maturation, and survival, promoting their recruitment to the esophageal mucosa [8]. IL-13 drives epithelial cell dysfunction, upregulates eotaxin-3 (CCL26), and orchestrates tissue remodeling and fibrosis [8]. Eotaxin-3 is a potent chemokine that establishes a chemotactic gradient for eosinophil migration and retention in the esophagus [9].

Genetic studies have identified risk loci for EoE in genes encoding epithelial proteins (e.g., CAPN14 (encodes a chief EoE susceptibility involved in regulating esophageal epithelial cell homeostasis) [10], DSG1 (loss of expression) [11], TSLP (polarization of Th2 immunity and induction of eotaxins) [12], STAT6, TMEM182, RAD50, SOX4, RHOG, RORA, MATN2, PRKG1, SHANK2, GPR12, GALNT1, SMAD3), highlighting the importance of epithelial barrier integrity and immune-epithelial crosstalk in disease susceptibility and pathogenesis [13].

The hallmark of EoE is epithelial barrier dysfunction, characterized by loss of tight junction proteins (e.g., E-cadherin, claudins, desmoglein-1), dilated intercellular spaces, and basal cell hyperplasia. IL-13 and CAPN14 are central to this process, with CAPN14 upregulated in the esophageal epithelium of EoE patients, contributing to proteolytic activity and further barrier disruption. The compromised barrier facilitates allergen penetration, perpetuating immune activation and chronic inflammation [14].

Eosinophils, the signature cell of EoE, release cytotoxic granule proteins—major basic protein (MBP), eosinophil cationic protein (ECP), eosinophil-derived neurotoxin (EDN), and eosinophil peroxidase (EPX)—which induce epithelial cell apoptosis, nerve dysfunction, and tissue injury. Eosinophil degranulation also promotes the formation of extracellular traps (EETs), amplifying local inflammation and tissue remodeling.

Mast cells are increased in EoE and release mediators such as histamine, tryptase, and TGF-β1, further exacerbating inflammation, smooth muscle dysfunction, and fibrosis. The interplay between eosinophils and mast cells is thought to be central to the pathogenesis of EoE and its complications [15].

Chronic inflammation in EoE leads to progressive tissue remodeling, characterized by subepithelial fibrosis, angiogenesis, smooth muscle hypertrophy, and epithelial–mesenchymal transition (EMT). Transforming growth factor-beta 1 (TGF-β1), upregulated in EoE, is a key mediator of fibroblast activation, extracellular matrix deposition, immune regulation, tissue repair, and fibrosis, resulting in esophageal stiffening, strictures, and reduced compliance [16]. Periostin, a matricellular protein induced by IL-13, further enhances fibrosis and tissue remodeling [17].

Notably, evidence from surgical specimens and animal models demonstrates that eosinophilic infiltration and fibrous remodeling can extend beyond the mucosa into the submucosal and muscular layers of the esophagus, paralleling mechanisms observed in other type 2 inflammatory diseases such as asthma and atopic dermatitis [18,19].

### 3.2. Mechanisms Linking EoE Inflammation to Extraesophageal (Laryngeal) Tissues

EoE frequently coexists with other atopic conditions, including asthma, allergic rhinitis, and atopic dermatitis, reflecting shared Th2-driven immunopathology. The “atopic march” concept posits that EoE may represent a late manifestation of systemic type 2 inflammation, with overlapping cytokine pathways (IL-4, IL-5, IL-13, TSLP) contributing to both esophageal and extraesophageal disease [2,3,7].

Several mechanisms have been proposed to explain how EoE-related inflammation might affect laryngeal tissues.

Direct extension of inflammation may occur in rare cases. Although eosinophilic infiltration is typically confined to the esophagus, available reports suggest that proximal involvement is uncommon but measurable in selected aerodigestive cohorts: in one pediatric series, laryngeal eosinophils were identified in 9 of 81 children (11%), and biopsy-proven EoE was present in 3 of those 9 children (33%) compared with 5 of 72 children without laryngeal eosinophils (6.9%) [4]. Other reports remain limited to isolated cases or small series involving hypopharyngeal or laryngopharyngeal lesions, so these findings should be interpreted as rare associations rather than established prevalence estimates, particularly in severe, refractory, or multidisciplinary airway-referral populations [20,21,22].

Indirect effects via cytokines and mediators may also contribute to laryngeal involvement. Even when direct eosinophilic infiltration is absent, cytokines and inflammatory mediators released during EoE may affect adjacent laryngeal tissues, contributing to symptoms such as cough, hoarseness, and subglottic stenosis [2,7].

Shared atopic predisposition further complicates interpretation of laryngeal findings. Patients with EoE often have concomitant atopic airway diseases, which can themselves produce laryngeal symptoms and lesions, making it difficult to distinguish EoE-related effects from comorbid conditions [3,7].

Reflux and microaspiration represent another potential pathway. EoE may be associated with an increased risk of gastroesophageal reflux, which can lead to laryngopharyngeal reflux (LPR) and microaspiration, thereby causing laryngeal injury and inflammation [20].

Murine models of EoE have demonstrated transmural inflammatory and fibrotic involvement, like the pathophysiology of bronchial asthma, supporting the plausibility of shared mechanisms affecting both esophageal and laryngeal tissues. Case reports and small series have described laryngeal manifestations such as subglottic stenosis, chronic cough, and croup in pediatric EoE patients, suggesting that, at least in some cases, EoE may contribute directly or indirectly to laryngeal pathology [22,23,24,25].

### 3.3. Clinical Manifestations Overlapping Between EoE and Laryngeal Disorders

EoE presents with a diverse range of symptoms that vary by age, disease stage, and comorbid conditions. In adults, dysphagia to solid foods, food impaction, chest pain, and refractory heartburn are hallmark symptoms [26]. In children, feeding difficulties, vomiting, regurgitation, abdominal pain, and failure to thrive are common and often contribute to diagnostic delay [26].

Importantly, EoE patients may develop adaptive behaviors (e.g., prolonged chewing, food avoidance, increased liquid intake) that mask underlying dysfunction and obscure diagnosis [25,26].

Several laryngeal and airway symptoms have been reported in EoE patients, including chronic cough, hoarseness and dysphonia, throat clearing, recurrent croup, stridor, subglottic stenosis, and recurrent pneumonia with chest congestion.

In pediatric populations, airway presentations may be prominent and sometimes precede or overshadow classic esophageal symptoms. Respiratory and aerodigestive symptoms, including cough, recurrent croup, and hoarseness, have been described in pediatric EoE cohorts; however, reported frequencies vary substantially according to referral setting, case definition, and whether children are recruited from gastroenterology, pulmonology, otolaryngology, or multidisciplinary aerodigestive clinics [26,27]. Therefore, these symptoms should be interpreted as clinically important associations rather than as evidence of a fixed prevalence or direct causal pathway, Table 1.

This table underscores the diagnostic complexity when laryngeal symptoms are present, as clinical features may overlap significantly among EoE, LPR, and VCD. The role of PPI therapy in LPR-like persistent throat symptoms should be interpreted cautiously: randomized placebo-controlled evidence has not shown a clear symptomatic benefit of empiric PPI therapy over placebo, so PPI response should not be rated as uniformly high. Children with EoE may present with airway-predominant symptoms, including chronic cough, recurrent croup, and even subglottic stenosis, sometimes in the absence of overt esophageal complaints. In a case–control study of children under 7 years with aerodigestive symptoms, food allergy and food pocketing were strong predictors of EoE, while liquid dysphagia and recurrent pneumonia were less likely to indicate EoE [27].

### 3.4. Eosinophilic Esophagitis in the Context of Eosinophilic Granulomatosis with Polyangiitis (EGPA)

In patients presenting with esophageal symptoms accompanied by marked peripheral hypereosinophilia (absolute eosinophil count [AEC] > 1500 cells/µL), additional evaluation should be considered to exclude disorders other than eosinophilic esophagitis (EoE), including non-EoE eosinophilic gastrointestinal diseases, hypereosinophilic syndrome (HES), and eosinophilic granulomatosis with polyangiitis (EGPA). Referral to an allergy/immunology specialist may facilitate the diagnostic evaluation and guide appropriate management [29]. EGPA, formerly known as Churg–Strauss syndrome, is a multisystem inflammatory disorder that occupies the intersection between eosinophilic diseases and anti-neutrophil cytoplasmic antibody (ANCA)-associated vasculitis. The 2022 ACR/EULAR classification criteria assign the following weighted scores: maximum peripheral eosinophil count ≥ 1 × 10^9^/L (+5 points), obstructive airway disease (+3 points), nasal polyps (+3 points), extravascular eosinophilic-predominant inflammation on biopsy (+2 points), and mononeuritis multiplex or motor neuropathy not attributable to radiculopathy (+1 point). Conversely, positivity for ANCA or anti-proteinase 3 (PR3)-ANCA (−3 points) and the presence of hematuria (−1 point) reduce the total score. A cumulative score of at least 6 points supports classification as EGPA in the appropriate clinical context [30].

The relationship between EGPA and laryngeal disorders is mainly indirect and should be distinguished from typical EoE-associated aerodigestive overlap. EGPA commonly involves the upper and lower airways through asthma, chronic rhinosinusitis, nasal polyposis, eosinophilic inflammation, and small-vessel vasculitis; laryngeal manifestations are less common but may occur as hoarseness, chronic cough, stridor, vocal fold edema or dysfunction, subglottic/tracheal stenosis, or vasculitic/eosinophilic lesions of the upper airway. Therefore, in a patient with EoE-like esophageal symptoms plus laryngeal complaints, marked peripheral eosinophilia, asthma, sinonasal disease, neuropathy, pulmonary infiltrates, or systemic inflammatory features should prompt consideration of EGPA rather than attributing the laryngeal disorder solely to EoE, LPR, or VCD. Conversely, isolated EoE with localized esophageal eosinophilia and nonspecific laryngeal symptoms does not by itself imply EGPA; objective evaluation for systemic vasculitis is needed before making that link [29,30].

### 3.5. Diagnostic Challenges: Distinguishing EoE from LPR and VCD

A fundamental challenge in EoE is the frequent discordance between patient-reported symptoms and histologic findings. Clinical trials of potent eosinophil-depleting agents (e.g., lirentelimab, benralizumab) have demonstrated robust histologic responses without corresponding symptomatic improvement, suggesting that eosinophils may not be the sole drivers of symptoms or tissue damage. This complicates both clinical management and trial design, as relying solely on eosinophil counts may not capture disease activity or therapeutic benefit [31].

Laryngopharyngeal Reflux (LPR) is characterized by the retrograde flow of gastric contents into the larynx and pharynx, causing symptoms such as hoarseness, cough, and throat clearing. LPR and EoE can present with similar symptoms, and both may coexist in some patients. Diagnostic tools for LPR include pH monitoring, laryngoscopy, and detection of pepsin in laryngeal tissue or bronchoalveolar lavage [20].

Vocal fold dysfunction (VCD), or paradoxical vocal fold motion, involves inappropriate adduction of the vocal folds during inspiration, leading to stridor, throat tightness, and dyspnea. VCD is often misdiagnosed as asthma or refractory airway disease and can coexist with EoE or be triggered by reflux, irritants, or psychological factors [5].

Endoscopy with biopsy remains the gold standard for EoE diagnosis, requiring ≥15 eosinophils per high-power field in esophageal biopsies. However, EoE is a patchy disease, and multiple biopsies from different esophageal segments are recommended to maximize diagnostic yield [32].

Consistent with current consensus guidance, PPI responsiveness is no longer used to distinguish PPI-responsive esophageal eosinophilia from EoE as a separate diagnostic entity. Patients with symptoms of esophageal dysfunction and esophageal eosinophilia meeting the histologic threshold are considered within the EoE spectrum once alternative causes of esophageal eosinophilia have been assessed, and PPIs are best framed as a therapeutic option rather than a diagnostic exclusion criterion [33].

Peripheral Blood and Noninvasive Biomarkers such as blood eosinophil counts, serum EDN, and other cytokines have been investigated but lack specificity and sensitivity for EoE, especially in distinguishing it from other atopic diseases [32,33].

Minimally Invasive Tools like the esophageal string test (EST), Cytosponge, and esophageal brushing have shown promise in detecting eosinophil-derived proteins and correlating with disease activity, but are not yet widely adopted in clinical practice [33,34,35].

Functional Testing using high-resolution manometry and EndoFLIP can assess esophageal motility and distensibility, providing insights into fibrostenotic disease and symptom generation [36].

Laryngoscopy and Pulmonary Function Testing are essential for evaluating laryngeal manifestations and VCD, but findings may be nonspecific and require correlation with clinical context [37].

Table 2 summarizes the level of evidence for the diagnostic tests available.

### 3.6. Endoscopic and Histologic Evaluation Relevant to Laryngeal Involvement

Characteristic endoscopic findings in EoE include rings (trachealization), which reflect subepithelial fibrosis and remodeling; linear furrows, which appear as crack-like fissures along the esophagus; white exudates, which represent eosinophilic microabscesses; and edema and strictures, which indicate advanced disease and reduced compliance.

The EoE Endoscopic Reference Score (EREFS) is a validated tool for quantifying these features and guiding biopsy site selection [43].

Multiple biopsies from at least two esophageal locations (distal and mid/proximal) are recommended due to the patchy nature of eosinophilic infiltration. Histologic features include a peak eosinophil count of ≥15 eos/hpf, basal cell hyperplasia, dilated intercellular spaces, eosinophilic microabscesses, and lamina propria fibrosis.

The EoE Histologic Scoring System (EoE-HSS) provides a comprehensive assessment of disease activity and differentiation from GERD [44].

High-Resolution Manometry (HRM) identifies motility disturbances, such as fragmented peristalsis and hypomotility, that are associated with inflammation and fibrosis [36].

EndoFLIP measures esophageal distensibility and compliance, correlating with fibrostenotic phenotype and symptom severity [37].

Imaging (Barium Esophagram, OCT) may reveal strictures, rings, and esophageal narrowing, but sensitivity is variable and not specific for EoE [45].

### 3.7. Evidence for Laryngeal Inflammatory Involvement Rather than General EoE Immunopathology

The mediator pathways described in Section 3.1 provide biological plausibility for laryngeal involvement, but direct evidence that eosinophil- or mast-cell-derived mediators injure laryngeal tissue in EoE remains limited. Rather than repeating the general immunopathology of EoE, this section focuses on whether those esophageal inflammatory pathways have been demonstrated in laryngeal or hypopharyngeal tissues and how strongly they support a causal link.

Direct evidence demonstrating eosinophilic inflammation in both esophageal and laryngeal tissues remains limited and is derived mainly from small pediatric aerodigestive cohorts and isolated case reports. In the best-described pediatric series, Yawn et al. evaluated 81 consecutive children referred for aerodigestive dysfunction who underwent microlaryngoscopy with posterior arytenoid biopsy, flexible bronchoscopy, esophagogastroduodenoscopy with esophageal biopsies, and reflux testing when clinically indicated. Laryngeal eosinophils were identified in 9 of 81 children (11%), with counts ranging from 1 to 29 eosinophils/high-power field; biopsy-proven EoE was present in 8 of 81 children overall, and 3 of the 9 children with laryngeal eosinophils also had biopsy-proven EoE. Thus, EoE was more frequent among children with posterior arytenoid eosinophils than among those without laryngeal eosinophils (33% versus 6.9%), suggesting an association but not proving causality [4]. A separate adult case report described a laryngopharyngeal lesion in a patient with established EoE in which laryngeal biopsy showed prominent inflammatory infiltrates but insignificant eosinophilia; later exacerbation of both the laryngopharyngeal lesion and EoE coincided with esophageal eosinophilia up to 46 eosinophils/high-power field, supporting temporal overlap rather than definitive shared tissue eosinophilia [20]. Taken together, these data indicate that true eosinophilic involvement of laryngeal tissue is uncommon, heterogeneous in degree, and incompletely linked to esophageal disease activity. The strongest available signal is that marked posterior arytenoid eosinophilia, particularly above 15 eosinophils/high-power field, may identify a subgroup with concomitant EoE, but larger prospective studies with standardized laryngeal biopsy criteria are needed.

Overall, these findings support biological plausibility but do not yet establish a direct causal pathway from esophageal eosinophil or mast-cell activation to laryngeal tissue injury. Future studies should therefore distinguish clearly between shared type 2 inflammatory susceptibility, reflux-mediated injury, comorbid atopic airway disease, and true laryngeal eosinophilic inflammation.

## 4. Discussion

This review was expected to clarify whether laryngeal manifestations in EoE are best understood as direct eosinophilic disease, secondary reflux-related injury, shared allergic inflammation, or overlapping conditions. Particular attention was given to reports of hoarseness, chronic cough, laryngitis, vocal fold changes, and subglottic stenosis, as these symptoms may prompt evaluation by otolaryngology before EoE is recognized.

Limitations included likely heterogeneity in diagnostic definitions, reliance on case reports, inconsistent use of laryngoscopy or biopsy, and possible publication bias toward unusual airway presentations. Nevertheless, this approach provides a transparent and reproducible method for summarizing current knowledge and identifying priorities for future research, including prospective studies that systematically assess laryngeal symptoms, endoscopic findings, reflux markers, and treatment response in patients with EoE.

### 4.1. ENT and Laryngology Perspectives on EoE

Otolaryngologists may encounter patients with aerodigestive symptoms that overlap with or prompt evaluation for EoE, especially in pediatric and multidisciplinary airway settings. In the single-centre retrospective series by Kubik et al., which included 251 patients with EoE, 18% initially presented to otolaryngology and 26% had airway symptoms. Patients diagnosed through ENT pathways were younger than those diagnosed through non-ENT pathways (mean age 3.4 versus 10.4 years) and were substantially more likely to have airway symptoms (68% versus 15%). These data suggest that ENT-facing presentations are clinically relevant, particularly in younger children, although the findings should be interpreted cautiously because they derive from a single-centre cohort rather than population-based prevalence data [46].

EoE has been implicated in cases of inflammatory subglottic stenosis, particularly in children with recurrent airway symptoms unresponsive to standard therapies [47].

Chronic cough and hoarseness may be the initial or predominant presentation in EoE, especially in pediatric populations [48].

Evidence linking EoE to failed laryngotracheal reconstruction or recurrent airway procedures remains limited and is derived mainly from retrospective aerodigestive cohorts, case series, and expert discussion rather than definitive causal studies. Accordingly, EoE should be considered as part of the broader preoperative evaluation in selected patients with unexplained, recurrent, inflammatory, or refractory airway disease—particularly when dysphagia, food impaction, feeding difficulty, atopy, or reflux-like symptoms are also present—rather than presented as a universal rule-out requirement before all surgical intervention [49].

A multidisciplinary approach involving gastroenterologists, allergists, dieticians, and otolaryngologists is essential for optimal diagnosis and management of EoE, particularly when extraesophageal manifestations are suspected. Early recognition and treatment can prevent irreversible complications such as fibrosis and strictures.

### 4.2. Evidence for Direct Causation: Case Reports and Clinical Trials

One study reports respiratory symptoms associated with EoE, including subglottic stenosis in selected pediatric cases, sometimes with airway-predominant presentation or without classic esophageal symptoms [50].

In a multidisciplinary pediatric cohort of children with chronic cough and recurrent croup, Greifer et al. reported histologic evidence of EoE in a subset of patients who underwent esophageal biopsy: 3 of 9 biopsied children (33%) had EoE, corresponding to 3 of the overall 25 children in the cohort (12%) [51]. These figures should be interpreted cautiously because biopsy was performed selectively rather than in all enrolled children. While direct eosinophilic infiltration of the larynx is rare, indirect effects via cytokines and mediators may contribute to laryngeal symptoms and lesions, Table 3.

### 4.3. Role of Reflux (Acid and Non-Acid) and Microaspiration in Laryngeal Findings

As defined in Section 3.5, LPR is an important competing or coexisting explanation for laryngeal symptoms in patients with EoE. In this context, the key issue is not the definition of LPR itself, but whether reflux burden, non-acid reflux, pepsin exposure, or microaspiration can account for laryngeal findings independently of EoE activity.

LPR and EoE share many clinical features, making differentiation challenging; therefore, diagnostic evaluation may require pH monitoring, impedance testing, and detection of pepsin or bile acids in laryngeal tissue or bronchoalveolar lavage to assess reflux and aspiration, although the sensitivity and specificity of these methods are variable. Reflux scintigraphy and EndoFLIP may provide additional information on reflux events and esophageal function [57].

EoE may increase the risk of reflux by impairing esophageal clearance and compliance, while reflux can exacerbate epithelial barrier dysfunction and promote eosinophil recruitment. The relationship is bidirectional and complex, with both conditions potentially contributing to laryngeal symptoms and lesions [58].

### 4.4. Vocal Fold Dysfunction and Functional Laryngeal Disorders in Patients with EoE

As defined in Section 3.5, VCD/paradoxical vocal fold motion is a functional laryngeal disorder that can mimic asthma or refractory airway disease. In patients with EoE, the main clinical challenge is determining whether VCD-like symptoms reflect primary inducible laryngeal obstruction, reflux-triggered laryngeal hyperresponsiveness, comorbid atopic airway disease, or EoE-associated aerodigestive overlap.

Laryngoscopy is the gold standard for diagnosis because it allows direct visualization of paradoxical vocal fold motion during symptomatic episodes. Pulmonary function testing may show a flattened inspiratory flow loop or an abnormal FEF50/FIF50 ratio, while provocative testing with exercise or irritant challenges can help elicit symptoms for diagnosis [59].

Patients with EoE may present with symptoms suggestive of VCD, and both conditions can coexist, particularly in those with refractory airway symptoms. Distinguishing between EoE-related laryngeal symptoms and primary VCD is essential for appropriate management [5].

In practice, EoE-related laryngeal symptoms and primary VCD or paradoxical vocal fold motion should be distinguished by combining symptom pattern, objective laryngeal visualization, esophageal history, and treatment response rather than by symptoms alone [5,58]. Primary VCD is suggested by episodic inspiratory dyspnea or stridor, throat tightness, exercise or irritant triggers, poor response to bronchodilators, and laryngoscopic evidence of paradoxical inspiratory vocal fold adduction during symptoms [58]. By contrast, EoE should be suspected in patients with VCD-like or refractory laryngeal complaints when symptoms coexist with dysphagia; food impaction; food avoidance; feeding difficulty or food pocketing in children; refractory reflux-like symptoms; strong atopic history; recurrent croup; chronic cough or hoarseness; subglottic stenosis; or failure of standard asthma, reflux, or speech-therapy-based VCD management [26,27,47,48,49,51]. Current evidence directly linking EoE to confirmed VCD is sparse; most available data concern aerodigestive symptoms and laryngeal manifestations more broadly [5,47,58]. Therefore, persistent or atypical VCD-like presentations should prompt multidisciplinary assessment, including laryngoscopy to document inducible laryngeal obstruction and gastroenterology evaluation with esophageal biopsies when EoE clues are present [5,32,47,58].

### 4.5. Pediatric Considerations: Airway-Predominant Presentations of EoE

Children with EoE may present with airway-predominant symptoms, including chronic cough, recurrent croup, stridor, and subglottic stenosis, sometimes in the absence of classic esophageal complaints. Early recognition is critical, as delayed diagnosis can lead to progression of fibrosis and irreversible complications.

Studies report a mean diagnostic delay of 49 months in children with aerodigestive symptoms, underscoring the need for heightened clinical suspicion. Food allergy and food pocketing are strong predictors of EoE in children with airway symptoms, while liquid dysphagia and recurrent pneumonia are less likely to indicate EoE [26,27,59].

### 4.6. Imaging and Functional Testing for Laryngeal Involvement

Flexible nasal endoscopy (FNE) remains the most practical first-line laryngeal test because it directly visualizes vocal fold mobility, mucosal inflammation, edema, suspected lesions, and signs of inducible laryngeal obstruction. However, FNE does not diagnose EoE and should be interpreted alongside targeted screening for esophageal symptoms, feeding history, atopy, and response to prior reflux, asthma, or speech-therapy-based interventions [37,47,58].

A practical step to shorten diagnostic delay is to incorporate brief EoE screening questions into the evaluation of unexplained laryngeal symptoms, especially asking about dysphagia, food impaction, slow eating, food avoidance, food pocketing, feeding difficulty, atopy, and prior failure of reflux or asthma therapy. When these clues are present, early gastroenterology referral for endoscopic assessment with esophageal biopsies should be favored rather than repeated empiric treatment of presumed LPR, asthma, or functional laryngeal disease alone [26,27,32,47,49,50].

To reduce diagnostic delay, authors should recommend a low-threshold referral pathway from ENT, pulmonology, allergy, and primary care to gastroenterology when laryngeal or airway symptoms are persistent, recurrent, unexplained, or refractory and coexist with EoE clues. Referral is particularly appropriate in patients with dysphagia, food impaction, slow eating, excessive chewing, avoidance of solid foods, food pocketing or feeding difficulty in children, vomiting or abdominal pain, strong atopic history, known food allergy, recurrent croup, chronic cough or hoarseness not responding to standard therapy, subglottic stenosis, failed or recurrent airway surgery, or persistent symptoms despite empiric reflux or asthma management [26,27,41,42,43,45,60,61].

The first-line gastroenterology evaluation for suspected EoE should remain upper endoscopy with multiple esophageal biopsies from different levels because EoE is patchy and histology is required for diagnosis. HRM, EndoFLIP, barium esophagram, pH-impedance monitoring, and other functional or imaging studies may be useful in selected patients, especially those with dysphagia out of proportion to endoscopic findings, suspected fibrostenotic disease, strictures, motility symptoms, or difficult reflux/LPR overlap. Nevertheless, HRM and EndoFLIP are not widely available and should not be presented as routine first-line tests when EoE is initially suspected; rather, they are complementary second-line tools used after or alongside endoscopic and histologic assessment in specialized centers [31,36,37,40,53,60].

### 4.7. Management Strategies When Laryngeal Manifestations Are Suspected in EoE Patients

Current pharmacologic treatments include PPIs, swallowed topical corticosteroids, dietary therapy, esophageal dilation for fibrostenotic disease, and dupilumab in eligible patients; these are the therapies most consistently reflected in contemporary guideline-based management, whereas cendakimab, benralizumab, mepolizumab, lirentelimab, etrasimod, and other biologic or small-molecule approaches remain investigational or not routinely guideline-recommended for EoE outside approved indications or clinical-trial settings [33,46,57,61]. The cited network meta-analysis should be interpreted as evidence of esophageal treatment efficacy, not direct causation of laryngeal disease: it compared pharmacologic therapies using dysphagia, histologic remission, endoscopic improvement, eosinophil counts, and histologic severity as outcomes, but it did not establish that EoE directly causes laryngeal manifestations or that these treatments improve objective laryngeal endpoints [46]. It also focused on adolescent and adult trial populations rather than pediatric-only evidence, so pediatric extrapolation should be cautious and should be aligned with pediatric guidelines [46,61]. In that analysis, 300 mg dupilumab, 360 mg cendakimab, and 2 mg budesonide oral suspension showed the strongest comparative efficacy signals across symptom, histologic, and endoscopic outcomes, with dupilumab showing the most consistent overall profile; however, only guideline-supported and approved therapies should be presented as current recommended options, while investigational agents should be explicitly labeled as such [46].

Proton Pump Inhibitors (PPIs) are first-line therapy for EoE and are effective in reducing eosinophil counts and improving symptoms in a subset of patients. Topical corticosteroids, including swallowed fluticasone or budesonide, are highly effective in inducing histologic remission and improving symptoms with minimal systemic absorption. Biologic agents, such as dupilumab (anti-IL-4/IL-13), are approved for EoE and other atopic diseases, offering a promising option for patients with coexisting airway involvement or refractory disease [57].

Elimination diets, including the six-food elimination diet (6FED) or stepwise elimination based on allergy testing, can achieve clinical and histologic remission in many patients. Elemental diets are reserved for refractory cases or for patients with multiple food allergies [58].

Esophageal dilation is indicated for patients with strictures or severe fibrostenotic disease and should be performed with caution because of the risk of perforation. Airway surgery may be required in cases of subglottic stenosis or recurrent airway obstruction. In selected patients with unexplained, recurrent, inflammatory, or refractory airway disease—especially when accompanied by atopy, dysphagia, feeding difficulty, food impaction, or reflux-like symptoms—evaluation for EoE before or during multidisciplinary airway planning may be appropriate, but current evidence does not support presenting this as a universal prerequisite for all laryngotracheal procedures [59].

When laryngeal lesions are suspected in a patient with known or possible EoE, management should follow a dual-track strategy: guideline-based control of esophageal inflammation and parallel objective evaluation of the laryngeal disorder. The first step is to characterize the airway presentation with ENT assessment, usually flexible laryngoscopy, documenting whether the finding is nonspecific edema/erythema, vocal fold dysfunction, granuloma, ulceration, recurrent croup, stridor, or subglottic stenosis. Severe, recurrent, obstructive, or surgically relevant disease may require microlaryngobronchoscopy, airway sizing, and biopsy only when clinically justified. At the same time, gastroenterology should confirm current EoE activity with symptom review, endoscopy with esophageal biopsies when indicated, and assessment of fibrostenotic disease, because laryngeal symptoms alone do not measure EoE activity [31,37,56,60,61,62]. If active EoE is present, treatment should use recommended EoE therapies—PPI, swallowed topical steroid, dietary elimination, dilation for strictures, and dupilumab in eligible refractory or atopic patients—but expected benefit should be framed as esophageal disease control, since laryngeal outcomes have not been validated as treatment endpoints [46,51,52,60,63,64]. In parallel, clinicians should actively evaluate and treat competing or coexisting causes of laryngeal injury, including LPR/GERD with pH-impedance testing when uncertainty persists, asthma or allergic rhinitis, chronic rhinosinusitis, infection, irritant exposure, and inducible laryngeal obstruction/VCD requiring speech–language therapy and breathing retraining [5,20,53,54]. For subglottic stenosis or fixed airway narrowing, airway-directed treatment such as endoscopic dilation, steroid injection, topical therapy, or reconstructive surgery should be individualized by airway severity and recurrence risk, while concurrently optimizing EoE and reflux/atopy control rather than assuming that EoE therapy alone will reverse the lesion [42,44,56,59]. Follow-up should reassess both compartments separately: esophageal symptoms, endoscopy, and histology for EoE activity, and repeat laryngoscopy/airway evaluation plus voice, cough, croup, stridor, or stenosis outcomes for laryngeal disease. This approach aligns the section title with the evidence: suspected laryngeal manifestations require multidisciplinary, phenotype-driven management, not simply escalation of EoE-directed therapy.

### 4.8. Biologic Therapies and Laryngeal Outcomes

Biologic therapies are discussed in Section 4.8 and summarized in Table 3. For laryngeal manifestations, their relevance remains indirect because available trials measured esophageal histology, endoscopy, and esophageal symptoms rather than objective airway outcomes. Benralizumab is a useful cautionary example: despite marked esophageal eosinophil depletion, it did not significantly improve dysphagia and did not evaluate hoarseness, cough, croup, stridor, vocal fold disease, or subglottic stenosis. Therefore, biologics should be presented as EoE-directed therapies with possible relevance to shared type 2 inflammation, not as established treatments for EoE-associated laryngeal lesions.

### 4.9. Guidelines and Consensus Statements Addressing Extraesophageal Manifestations of EoE

Recent guidance from major gastroenterology and allergy/pediatric societies should be cited explicitly. The 2025 ACG Clinical Guideline for diagnosis and management of EoE, the 2026 ASGE consensus recommendations on endoscopic management, and the 2024 ESPGHAN pediatric guideline all emphasize that diagnosis requires symptoms of esophageal dysfunction plus esophageal biopsies, with multiple biopsies from different esophageal levels because of patchy disease; they also support multidisciplinary care and guideline-based anti-inflammatory therapy, dietary therapy, dilation when indicated, and maintenance treatment [60,61,65]. Allergy-focused guidance in the manuscript, including the EAACI Task Force report on feeding difficulties, is most relevant for recognizing pediatric feeding and atopic clues that may prompt EoE evaluation in children with aerodigestive or laryngeal complaints [66]. However, these guidelines do not define laryngeal manifestations as diagnostic criteria or standard treatment endpoints for EoE. Therefore, in patients with refractory aerodigestive symptoms, recurrent croup, chronic cough, hoarseness, or suspected airway lesions, the guideline-consistent recommendation is targeted EoE evaluation when compatible esophageal, feeding, reflux-like, or atopic features are present, combined with parallel ENT assessment, rather than assuming direct laryngeal involvement [60,61,64,65].

Minimally invasive and noninvasive EoE monitoring tools are summarized in Section 3.5 and Table 2, but they should not be considered as established diagnostic methods. Current pediatric and adult guidelines continue to emphasize upper endoscopy with multiple esophageal biopsies as the diagnostic standard, while tools such as Cytosponge, esophageal string test, esophageal brushing, peripheral biomarkers, and FeNO remain nonstandardized, validation-cohort-dependent, and not guideline-recommended as routine substitutes for endoscopy/biopsy. In the context of laryngeal manifestations, these methods may be useful for research or selected monitoring of esophageal inflammatory activity, but they do not diagnose laryngeal involvement, do not replace objective ENT assessment, and should be clearly labeled as adjunctive or investigational rather than recommended diagnostic tools.

While direct evidence is limited, case series and aerodigestive-clinic reports suggest that unrecognized EoE may coexist with recurrent laryngeal or airway lesions in some patients. Rather than establishing causation or a universal surgical protocol, these data support targeted consideration of EoE evaluation in patients with recurrent or refractory airway disease when compatible esophageal, feeding, reflux-like, or atopic features are present [67].

For patients undergoing laryngotracheal surgery, current evidence supports targeted rather than universal preoperative evaluation for EoE. Screening should be considered before airway reconstruction, repeat endoscopic dilation, or surgery for inflammatory/recurrent subglottic stenosis when there are compatible clues such as dysphagia, food impaction, slow eating, food avoidance, food pocketing, feeding difficulty, vomiting, refractory reflux-like symptoms, strong atopic history, food allergy, chronic cough, recurrent croup, hoarseness, unexplained airway inflammation, or prior failed airway intervention. A practical preoperative pathway would include structured symptom and feeding-history screening by ENT/pulmonology, review of atopy and reflux history, gastroenterology referral when EoE clues are present, and upper endoscopy with multiple esophageal biopsies if clinically indicated. Reflux assessment with pH-impedance monitoring may be useful when LPR/GERD is suspected or when reflux control could affect surgical outcomes. If EoE is confirmed, anti-inflammatory treatment should be optimized before or in parallel with airway surgery when timing permits; however, airway procedures should not be delayed in urgent obstruction. The goal is not to make EoE a mandatory rule-out diagnosis before all laryngotracheal operations, but to identify modifiable esophageal inflammation, reflux overlap, and atopic comorbidity that may contribute to persistent airway inflammation, restenosis, or poor postoperative response [44,56,59,60,61,68].

Feeding difficulties are relevant to laryngeal involvement because they may signal pediatric EoE in children whose first presentation is aerodigestive rather than classic dysphagia. In this setting, food refusal, food pocketing, slow eating, choking, or texture avoidance should be interpreted as clinical clues that may coexist with chronic cough, recurrent croup, hoarseness, or suspected aspiration. Current evidence remains heterogeneous and lacks standardized feeding-difficulty definitions, but these symptoms support targeted EoE screening when laryngeal or airway complaints are persistent, recurrent, or refractory [69].

Future research in eosinophilic esophagitis (EoE) should focus on addressing several important knowledge gaps to improve patient outcomes. Priorities include conducting comparative effectiveness studies of first-line therapies and evaluating biologic treatments in patient populations that have been underrepresented in clinical trials, such as newly diagnosed individuals, proton pump inhibitor (PPI)-naïve patients, and those with severe fibrostenotic disease. Research should also aim to identify predictors of treatment response and incorporate these into personalized treatment strategies. Further work is needed to define EoE phenotypes and endotypes associated with progression to fibrostenosis and to validate the clinical application of the Index of Severity in EoE (I-SEE) for treatment selection and disease monitoring. Additional priorities include developing reliable methods to identify food triggers, discovering noninvasive biomarkers, and expanding the use of minimally invasive techniques for monitoring therapeutic response. Future studies should also investigate the role of combination therapies, establish evidence-based dose reduction strategies, define quality indicators for EoE care, assess the cost-effectiveness of available treatments, and determine the optimal integration of emerging therapies into the EoE treatment algorithm. Collectively, these research efforts are expected to support more personalized, effective, and cost-efficient management of EoE [70].

## 5. Limitations of the Scoping Review

This scoping review has several limitations that should be considered when interpreting its findings. First, although the review was designed to map the available evidence on the relationship between eosinophilic esophagitis and laryngeal manifestations, the evidence base remains limited, heterogeneous, and largely observational. Much of the literature consists of case reports, small case series, retrospective cohort studies, and narrative reviews, which restricts the ability to establish causality or estimate the true prevalence of laryngeal involvement among patients with EoE. The rarity of reported laryngeal manifestations, especially direct eosinophilic infiltration of laryngeal or hypopharyngeal tissue, may reflect either genuinely uncommon pathology or under-recognition due to inconsistent evaluation by otolaryngology. Second, there was substantial variability in diagnostic definitions and assessment methods across studies. EoE diagnostic criteria have evolved over time, particularly with respect to the role of proton-pump inhibitor responsiveness, and older studies may not align fully with contemporary consensus definitions. Similarly, laryngeal findings were often described using nonspecific terms such as edema, erythema, laryngitis, or airway inflammation, without standardized laryngoscopic scoring, histologic confirmation, or objective reflux testing. This limits comparison across studies and makes it difficult to distinguish EoE-related laryngeal pathology from laryngopharyngeal reflux, vocal fold dysfunction, asthma, allergic rhinitis, infection, irritant exposure, or other comorbid aerodigestive conditions. Third, PubMed/MEDLINE was the only formal bibliographic database searched. Although 18 additional records were identified through supplementary sources, including reference-list screening and targeted searches for specific airway, laryngeal, and aerodigestive presentations, relevant otolaryngology, pediatric-airway, conference, interdisciplinary aerodigestive, or grey-literature reports indexed in Embase, Scopus, Web of Science, or Cochrane CENTRAL may have been missed. This introduces the possibility of database bias, language bias, and publication bias, particularly because unusual airway manifestations are more likely to be published than negative or routine clinical findings. Fourth, the protocol was registered in OSF under the identifier 9g6kc, but amendments made during revision, including expanded reporting of the search strategy and evidence-map characteristics, should be interpreted as clarifications of reporting rather than changes to the review question. Fifth, because this was a scoping review rather than a systematic review focused on intervention effectiveness, formal quality appraisal was not applied uniformly to all included sources. As a result, the strength of individual studies varied, and findings should be interpreted as a map of existing knowledge rather than definitive evidence for clinical decision-making. Sixth, pediatric and adult populations were included together because of the limited number of studies, but airway-predominant symptoms, diagnostic pathways, endoscopic practices, and treatment responses may differ substantially by age. This may reduce the precision of conclusions for specific patient groups. Seventh, treatment outcomes were inconsistently reported, particularly for laryngeal symptoms after EoE-directed therapy, making it difficult to determine whether improvement resulted from control of esophageal eosinophilia, reflux suppression, management of comorbid atopy, speech therapy, surgical intervention, or natural disease variation. Finally, the review highlights an important gap in prospective, multidisciplinary research. Future studies should use standardized EoE criteria, structured laryngoscopic assessment, esophageal and airway symptom instruments, reflux monitoring, histologic sampling when appropriate, and longitudinal follow-up to clarify whether laryngeal manifestations represent direct eosinophilic disease, secondary reflux-mediated injury, shared type 2 inflammation, or coincidental comorbidity, Table 4.

## 6. New Analyses and Research Suggestions

The novelty of this review lies in its explicit focus on laryngeal manifestations and airway-predominant presentations rather than on EoE as a general topic for otolaryngologists. Previous otolaryngology-oriented reviews have usefully introduced EoE to ENT practice and summarized common aerodigestive presentations [41,66]. In contrast, the present scoping review adds a structured evidence map that separates possible direct laryngeal eosinophilic disease from indirect mechanisms such as shared type 2 inflammation, reflux or microaspiration, and comorbid atopic airway disease; distinguishes pediatric airway-predominant evidence from adult/adolescent EoE evidence; compares esophageal diagnostic and monitoring tools with laryngeal assessment methods; and proposes specific research designs, including pediatric case–control studies of subglottic stenosis, prospective multidisciplinary registries, and biomarker studies linking esophageal inflammatory activity with objective laryngeal findings.

Several targeted research directions emerge from the gaps identified in this scoping review. First, a prospective multicenter registry should be developed for children and adults with confirmed eosinophilic esophagitis who have laryngeal or aerodigestive symptoms, or who are evaluated in combined gastroenterology–otolaryngology settings. Such a registry should use contemporary EoE diagnostic criteria, standardized esophageal phenotyping, and uniform laryngeal assessment across participating centers. At enrollment, patients should undergo structured symptom collection, endoscopic EoE scoring, histologic assessment from multiple esophageal levels, and standardized laryngeal evaluation using flexible nasal endoscopy when clinically appropriate. In patients with more severe airway symptoms, suspected subglottic disease, recurrent croup, stridor, failed airway surgery, or unclear laryngeal pathology, microlaryngobronchoscopy could provide more detailed airway characterization. Reflux testing, ideally using pH-impedance monitoring when feasible, should be incorporated to help distinguish acid or non-acid reflux-mediated laryngeal injury from EoE-associated inflammation. Optional laryngeal or hypopharyngeal biopsy could be considered only when clinically justified and ethically appropriate, particularly in cases with visible mucosal lesions or unexplained stenosis. The main aims of this registry would be to estimate the prevalence of laryngeal abnormalities in EoE, define clinical phenotypes, identify predictors of airway involvement, and evaluate outcomes after EoE-directed therapy, reflux management, dietary intervention, biologic treatment, or airway procedures. Longitudinal follow-up would be essential to determine whether laryngeal findings improve with histologic remission of EoE, persist independently, or correlate more closely with reflux burden or comorbid atopic airway disease.

Second, a dedicated pediatric case–control study is warranted to clarify the association between subglottic stenosis and EoE. This study could compare children with confirmed subglottic stenosis, including idiopathic, inflammatory, recurrent, or post-reconstruction stenosis, with matched controls without subglottic stenosis. Matching variables should include age, sex, atopic history, asthma, allergic rhinitis, prior intubation, reflux symptoms, and relevant comorbidities. All participants should undergo systematic screening for EoE symptoms and, when clinically indicated, standardized upper endoscopy with esophageal biopsies. The primary outcome would be the prevalence of EoE among children with subglottic stenosis compared with matched controls. Secondary outcomes could include stenosis severity, number of airway interventions, restenosis or recurrence rates, response to EoE-directed treatment, need for reflux therapy, and quality-of-life measures. This design would help determine whether EoE is simply an associated atopic condition in children with complex airway disease or whether it represents a modifiable contributor to inflammatory airway narrowing and poor surgical outcomes.

Third, biomarker correlation studies should pair minimally invasive EoE monitoring tools with standardized laryngeal findings to explore whether esophageal inflammatory activity can be linked noninvasively to airway manifestations. The esophageal string test and Cytosponge are particularly relevant because they can capture eosinophil-derived proteins, inflammatory mediators, and epithelial markers that may correlate with disease activity while reducing reliance on repeated endoscopy. Future studies should collect EST or Cytosponge samples alongside esophageal biopsies, symptom scores, laryngoscopic findings, reflux testing results, and treatment outcomes. Candidate biomarkers could include eosinophil-derived neurotoxin, major basic protein, eosinophil peroxidase, eotaxin-3, periostin, epithelial barrier markers, and type 2 cytokine signatures. Analyses should test whether biomarker patterns correlate with specific laryngeal phenotypes such as edema, erythema, vocal fold changes, recurrent croup, chronic cough, or subglottic stenosis. If validated, these approaches could support a less invasive framework for identifying patients whose laryngeal symptoms are likely to reflect active EoE biology rather than isolated reflux, functional laryngeal disorder, or unrelated airway pathology.

## 7. Conclusions

The relationship between eosinophilic esophagitis and laryngeal manifestations is multifaceted, reflecting complex immune mechanisms, shared atopic predisposition, and overlapping clinical presentations. While direct eosinophilic infiltration of the larynx is rare, EoE can contribute to laryngeal symptoms and lesions via indirect effects of cytokines, mediators, and comorbid atopic diseases. Diagnostic challenges abound, given the symptom overlap with LPR and VCD, the patchy nature of EoE, and the limitations of current diagnostic tools.

A high index of suspicion, comprehensive evaluation—including endoscopy with multiple biopsies, functional testing, and, when appropriate, noninvasive biomarkers—and multidisciplinary management are essential for optimal care. Early recognition and treatment of EoE in patients with laryngeal symptoms can prevent irreversible complications, improve quality of life, and enhance outcomes of airway interventions.

Ongoing research into the immunopathology of EoE, the development of noninvasive diagnostic tools, and the integration of biologic therapies targeting shared inflammatory pathways hold promise for advancing the care of patients with EoE and associated laryngeal manifestations. As our understanding of the interplay between esophageal and laryngeal inflammation deepens, so too will our ability to provide personalized, effective, and holistic care for this complex patient population.

## Figures and Tables

**Table 1:** Overlapping clinical features of EoE, LPR, and VCD.

Feature	EoE [2,7,14]	LPR [5,20]	VCD [28]
Primary Symptoms	Dysphagia, food impaction	Throat clearing, hoarseness, cough	Inspiratory stridor, throat tightness
Inflammatory Cells	Eosinophils, mast cells	Neutrophils, pepsin-mediated injury	No inflammation
Triggering Factors	Food allergens, aeroallergens	Acid reflux, dietary triggers	Exercise, stress
Diagnostic Tools	Endoscopy + biopsy (≥15 eos/hpf)	pH monitoring, laryngoscopy	Laryngoscopy with paradoxical motion
Role of PPI Therapy	Therapeutic option; response does not exclude EoE	Variable/limited; randomized evidence does not support empiric high response in persistent throat symptoms	Minimal
Response to Steroids	High (topical steroids effective)	Variable	Often unresponsive
Associated Conditions	Asthma, allergic rhinitis, eczema	GERD	Asthma, anxiety

**Table 2:** Diagnostic tools for EoE diagnosis/monitoring and evaluation of laryngeal involvement, highlighting their different clinical targets.

Diagnostic Tool	Primary Diagnostic Target	Main Finding Assessed	Sensitivity/Specificity	Notes
Endoscopy with Biopsy [14,32]	EoE diagnosis	Esophageal eosinophil density and histologic activity	Gold standard	Guideline-supported diagnostic standard for EoE; confirms esophageal disease rather than laryngeal manifestations
Peripheral Blood Tests [33,38]	EoE support/phenotyping	Systemic eosinophilia and cytokine activity	Variable	Adjunctive only; not recommended as a standalone diagnostic test because of limited specificity and overlap with other atopic diseases
Cytosponge [35,36,38]	EoE monitoring	EDN and esophageal eosinophil-related markers	75%/86% in a two-center prospective cross-sectional study of known EoE patients undergoing Cytosponge followed by endoscopy/biopsy; 105 successful procedures in 80 patients, with biopsy ≥ 15 eos/hpf as the reference standard [39]	Promising monitoring tool but not guideline-recommended as a replacement for endoscopy/biopsy; evidence remains validation-cohort-dependent and it does not directly assess the larynx
Esophageal String Test [35,37]	EoE monitoring	EDN, MBP-1, EPX, eotaxin-3, CLC	Biomarker levels correlated with peak eosinophils/hpf in pediatric pilot data and in a prospective multisite study; no single sensitivity/specificity estimate should be generalized [36]	Adjunctive/investigational monitoring method; not guideline-recommended for routine diagnosis and not a structural airway assessment
Esophageal Brushing (NGT) [35,36,39]	EoE monitoring	EDN	Blind NGT brushing using EDN ≥ 10 µg/mL in a prospective pediatric/young-adult validation study; 209 brushing samples from 94 patients, including active EoE, GERD, EoE remission, and no-disease controls [39]	Emerging monitoring approach; not yet a guideline-recommended routine diagnostic substitute for endoscopy/biopsy and samples esophageal rather than laryngeal tissue
Exhaled Breath (FeNO) [37,39]	Airway/laryngeal inflammatory context	Exhaled nitric oxide	Higher FeNO in pediatric EoE versus controls, but only weak correlation with esophageal eosinophils; adjunctive, not diagnostic alone [40]	Adjunctive airway inflammatory marker; not validated or recommended as a standalone EoE diagnostic test
Multichannel pH Impedance [14,32]	Reflux/laryngeal overlap	Acid/non-acid reflux burden and baseline impedance	Not correlated with eosinophil count	Useful when reflux/LPR overlap is suspected; does not diagnose EoE and should be interpreted with symptoms, endoscopy, and laryngeal findings
Flexible Laryngoscopy/FNE [37,41]	Laryngeal involvement	Laryngeal structure and vocal fold function	Variable	Recommended clinical tool for evaluating laryngeal anatomy/function, but findings are nonspecific and do not diagnose EoE
Microlaryngobronchoscopy [42]	Laryngeal/airway involvement	Subglottic stenosis, airway lesions, tissue pathology	Context-dependent	Reserved for selected severe, recurrent, or refractory airway presentations; not a routine EoE diagnostic method, but may allow airway assessment and biopsy when clinically justified

The most important distinction is that EoE tools primarily document esophageal inflammation or remodeling, usually through histology or esophageal biomarkers, whereas laryngeal tools directly assess airway anatomy, vocal fold function, and visible mucosal lesions. Only endoscopy with esophageal biopsy should be presented as the guideline-supported diagnostic standard for EoE. Peripheral blood tests, Cytosponge, esophageal string test, esophageal brushing, and FeNO should be framed as adjunctive, emerging, or investigational tools rather than validated substitutes for guideline-recommended diagnosis; their reported performance depends on specific study cohorts and reference standards. Similarly, laryngoscopy and microlaryngobronchoscopy evaluate laryngeal involvement but do not diagnose EoE. Reflux testing occupies an intermediate role because it helps determine whether laryngeal findings are reflux-mediated rather than directly related to EoE activity.

**Table 3:** Summary of key clinical trials and case reports.

Study/Report	Patient Number	Population	Intervention/Observation	Key Findings
Cooper et al.	80 patients (10 subglottic stenosis, 7 laryngeal clefts, 6 subglottic hemangiomas, 4 tracheomalacia, 3 laryngomalacia)	80 children with atypical croup	Esophageal biopsy	6.25% had >15 eos/hpf, suggestive of EoE and >1 in 10 among those in whom EoE was sought [52]
KRYPTOS trial	276 patients (51 adolescents)	Adolescents/adults	Lirentelimab (anti-Siglec-8)	Esophageal histologic remission/response refers to esophageal biopsy eosinophil reduction/remission, not laryngeal tissue. The trial enrolled 276 patients, including 51 adolescents; reported symptom outcomes were EoE/esophageal symptoms, mainly dysphagia, with only modest improvement. Laryngeal symptoms or objective laryngeal findings were not prespecified or reported as endpoints, so the trial did not establish benefit for laryngeal manifestations [53].
MESSINA trial	211 patients (28 patients age 12–17)	Adolescents/adults	Benralizumab (anti-IL-5Rα)	Benralizumab strongly reduced esophageal eosinophilia in MESSINA (histologic response 87.4% vs. 6.5% with placebo at week 24), but did not significantly improve dysphagia versus placebo and did not assess laryngeal symptoms, laryngoscopy, or airway endpoints. Its relevance here is therefore limited to showing that eosinophil depletion improves esophageal histology but does not establish benefit for laryngeal manifestations [54].
Dupilumab trials (LIBERTY EoE TREET Study, Chehade et al.)	Patients ≥ 12 years [55] Patients 1–11 years [56]	Adults/adolescents	Anti-IL-4/IL-13	Dupilumab trials assessed esophageal histologic, endoscopic, and symptom outcomes. Histologic remission/response refers to esophageal biopsy improvement, not laryngeal histology. In patients ≥ 12 years, dupilumab improved esophageal histologic and dysphagia-related outcomes in the LIBERTY EoE TREET program; in children 1–11 years, high-dose dupilumab achieved esophageal histologic remission in 68% versus 3% with placebo at week 16. Reported symptoms were EoE/esophageal or feeding-related symptoms, not laryngeal manifestations, and no trial demonstrated objective improvement in hoarseness, cough, croup, stridor, vocal fold disease, or subglottic stenosis [55,56].

**Table 4:** Age-specific evidence contributing to this scoping review and implications for interpretation.

Evidence Domain	Pediatric Evidence	Adult/Adolescent Evidence
Airway-predominant presentations	Most directly represented. Pediatric aerodigestive cohorts and case–control studies describe chronic cough, choking, recurrent croup, hoarseness, stridor, subglottic stenosis, food allergy, food pocketing, and feeding difficulty as clinically relevant clues to EoE [19,23,27,41,45,48].	Less directly represented. Adult EoE studies usually focus on dysphagia, food impaction, fibrostenosis, reflux-like symptoms, and treatment response; laryngeal outcomes are rarely prespecified or systematically measured [25,46,49,50,51].
Objective laryngeal/airway evaluation	Available mainly through multidisciplinary aerodigestive pathways, including flexible laryngoscopy, bronchoscopy, triple endoscopy, posterior arytenoid biopsy in selected cohorts, and evaluation of recurrent croup or chronic cough [4,19,23,41,43,45].	Sparse. Adult evidence includes isolated laryngopharyngeal case reports and otolaryngology-oriented reviews, but routine laryngoscopy, laryngeal biopsy, and reflux/airway phenotyping are uncommon in adult EoE trials [19,41,43].
Association with EoE diagnosis	Evidence supports targeted suspicion rather than prevalence estimates: refractory aerodigestive cohorts report a minority with biopsy-confirmed EoE, recurrent croup cohorts identify EoE or esophagitis in selected patients, and food allergy/food pocketing may help prioritize endoscopic evaluation [19,23,27,45,48].	Evidence supports biological plausibility and clinical overlap more than causal inference. Adult case reports show temporal overlap between EoE and laryngopharyngeal disease, while adult trials establish EoE treatment effects on esophageal outcomes, not airway-specific outcomes [20,46,49,50,51].
Treatment-response evidence	Often anecdotal or retrospective. Pediatric reports suggest that recognizing and treating EoE may be relevant in selected refractory airway cases, but improvement in laryngeal symptoms is not consistently separated from reflux therapy, diet, topical steroids, atopy management, or airway procedures [41,42,43,59,61].	Stronger for esophageal histologic and symptomatic outcomes than for laryngeal outcomes. Biologic and steroid trials demonstrate EoE efficacy in adults/adolescents, but they do not determine whether airway-predominant symptoms improve with EoE remission [46,49,50,51,64].
Implication for this review	Pediatric data drive the airway-focused conclusions and justify emphasizing recurrent croup, chronic cough, feeding clues, atopy, and subglottic disease as prompts for multidisciplinary EoE evaluation.	Adult data should be used mainly to support general EoE biology, diagnostic standards, and treatment principles; extrapolation to airway-predominant presentations should be cautious.

## Data Availability

No new data were created or analyzed in this study. Data sharing is not applicable.

## Supplementary Materials

The following supporting information can be downloaded at: https://www.mdpi.com/article/10.3390/jcm15166451/s1, Table S1: The initial 38 articles included in the analysis.

## Abbreviations

**Abbreviation**	**Definition**
6FED	Six-food elimination diet
CCL26	C-C motif chemokine ligand 26; eotaxin-3
CLC	Charcot–Leyden crystal protein
ECP	Eosinophil cationic protein
EDN	Eosinophil-derived neurotoxin
EETs	Eosinophil extracellular traps
EMT	Epithelial–mesenchymal transition
ENT	Ear, nose, and throat
EoE	Eosinophilic esophagitis
EoE-HSS	Eosinophilic Esophagitis Histologic Scoring System
EPX	Eosinophil peroxidase
EREFS	Eosinophilic Esophagitis Endoscopic Reference Score
EST	Esophageal string test
FeNO	Fractional exhaled nitric oxide
FNE	Flexible nasal endoscopy
GERD	Gastroesophageal reflux disease
HRM	High-resolution manometry
IL	Interleukin
ILC2	Group 2 innate lymphoid cell
LPR	Laryngopharyngeal reflux
MBP	Major basic protein
MLB	Microlaryngobronchoscopy
OCT	Optical coherence tomography
PPI	Proton-pump inhibitor
PRISMA-ScR	Preferred Reporting Items for Systematic Reviews and Meta-Analyses extension for Scoping Reviews
TGF-β1	Transforming growth factor-beta 1
Th2	Type 2 helper T-cell immune response
TSLP	Thymic stromal lymphopoietin
VCD	Vocal fold dysfunction

## Appendix A

**Table A1:** PRISMA-ScR checklist for this scoping review.

Item	Topic	PRISMA-ScR Reporting Item	Where Reported/Manuscript Detail
1	Title	Identifies the report as a scoping review.	Title: relationship between EoE and laryngeal manifestations; manuscript describes itself as a scoping review in Methods.
2	Abstract	Provides a structured summary of background, objectives, eligibility, information sources, charting, results, and conclusions.	Abstract includes Background, Methods, Results, and Conclusions; it summarizes PubMed/MEDLINE searching, included evidence types, mapped mechanisms, limitations, and future directions.
3	Rationale	Describes the rationale for the review in the context of what is already known.	Section 1 explains the clinical overlap between EoE, laryngeal symptoms, LPR, VCD, recurrent croup, chronic cough, and subglottic stenosis.
4	Objectives	States the review questions and key elements of population, concept, and context.	Section 1 and Section 2 state the question: what evidence links EoE with laryngeal pathology, mechanisms, diagnostic approaches, treatments, and research gaps.
5	Protocol	Indicates whether a protocol exists and where it can be accessed.	Section 2 reports OSF registration identifier 9g6kc; Section 5 explains that later additions clarified reporting rather than changing the review question.
6	Eligibility criteria	Specifies characteristics of sources used as criteria for eligibility and rationale.	Section 2 includes human English-language case reports, case series, observational studies, clinical trials, and reviews; excludes animal-only reports, non-English studies, and conference abstracts without extractable data.
7	Information sources	Describes all information sources and date of last search.	Section 2 reports PubMed/MEDLINE searched on 1 March 2026 plus supplementary reference-list and targeted searches; Section 5 notes database limitations.
8	Search	Presents the full electronic search strategy for at least one database.	Section 2 gives the full PubMed/MEDLINE string combining EoE terms with larynx, laryngitis, LPR, subglottic stenosis, vocal fold, dysphonia, hoarseness, cough, croup, stridor, and aerodigestive terms.
9	Selection of sources	States the process for selecting sources of evidence.	Section 2 describes deduplication, independent title/abstract screening, full-text review, and disagreement resolution by discussion or a third reviewer.
10	Data charting	Describes the data-charting process and whether forms were piloted or updated.	Section 2 reports use of a standardized extraction form for citation data, country, design, age group, atopy, EoE criteria, biopsies, laryngeal symptoms, laryngoscopy, reflux testing, treatment, and outcomes.
11	Data items	Lists and defines variables for which data were sought.	Section 2 and Table 1, Table 2, Table 3 and Table 4 chart clinical manifestations, diagnostic tools, laryngeal findings, treatment evidence, age group, and evidence limitations.
12	Critical appraisal	If done, describes appraisal methods and how appraisal informed synthesis.	Section 2 states that uniform formal appraisal was not required for this scoping review; higher-level studies were interpreted with appropriate tools when relevant. Section 5 lists this as a limitation.
13	Synthesis	Describes methods of handling and summarizing charted data.	Section 3, Section 4, Section 5 and Section 6 use narrative synthesis organized by pathophysiology, diagnostic overlap, laryngeal evaluation, treatment, limitations, and research priorities.
14	Selection results	Reports numbers screened, assessed, excluded, and included, ideally with a flow diagram.	Figure 1 and accompanying text report 330 records identified, 314 screened after duplicates, 67 full texts assessed, and 38 sources included.
15	Characteristics	Presents characteristics of included sources and citations.	Figure 1 text describes included designs; Table 3 and Table 4 summarize clinical trials, case reports, pediatric studies, and adult/adolescent evidence.
16	Critical appraisal results	If done, presents appraisal results.	Not applicable as a uniform exclusion criterion; limitations and study-design constraints are discussed in Section 5 and reflected in Table 4.
17	Individual results	Presents relevant data from each included source.	Table 2, Table 3 and Table 4 and Section 3 and Section 4 present mapped findings for diagnostic tools, laryngeal symptoms, direct/indirect mechanisms, and treatment evidence.
18	Synthesis results	Summarizes charted results in relation to review objectives.	Section 3, Section 4, Section 5 and Section 6 synthesize evidence on mechanisms, differential diagnosis, pediatric airway presentations, laryngeal assessment, treatment gaps, and future study designs.
19	Summary of evidence	Summarizes main results and relevance to clinicians, policy, or research.	Section 4, Section 6 and Section 7 summarize that direct laryngeal eosinophilia is uncommon, indirect pathways are plausible, and pediatric airway presentations warrant targeted multidisciplinary evaluation.
20	Limitations	Discusses limitations of evidence sources and the review process.	Section 5 addresses heterogeneity, small observational evidence, inconsistent laryngoscopy/biopsy and reflux testing, PubMed-only formal search, language restriction, and pediatric/adult pooling.
21	Conclusions	Provides a general interpretation of results and implications.	Section 7 concludes that EoE should be considered in persistent or recurrent laryngeal symptoms, especially when refractory or associated with atopy, and calls for standardized prospective research.
22	Funding	Describes sources of funding and role of funders.	Funding statement reports no external funding; acknowledgments identify institutional publication support.
