# Supplementary material for: The Relationship Between Eosinophilic Esophagitis and Laryngeal Manifestations: Pathophysiology, Clinical Overlap, and Diagnostic Challenges
Source: J Clin Med. 2026 Aug 20;15(16):6451. doi: 10.3390/jcm15166451 (PMC13514315; doi:10.3390/jcm15166451)
Supplement: Supplementary file 1 [file jcm-15-06451-s001.zip › jcm-4434856-supplementary.pdf]

Table S1: The initial 38 articles included in the analysis.

Number	Author	Year	Design	n	Age group	EoE criteria	Laryngeal assessment	Findings	Reflux testing	Outcome
**1**	**González-Cervera et al. [3]**	2017	Systematic review & meta-analysis	21 studies	Mixed	Standard histologic criteria (≥15 eos/hpf)	None	Strong association between EoE and atopic diseases (asthma, AR, eczema, FA)	None	Quantified atopic overlap; supports shared immunologic pathways
**2**	**Yawn et al. [4]**	2015	Case series	10 children	Pediatric	Eosinophilic infiltration of larynx + esophageal evaluation	Direct laryngeal exam; biopsy	Eosinophilic laryngitis identified in aerodigestive dysfunction; chronic cough/dysphonia	Some patients underwent reflux evaluation	Symptom improvement with targeted therapy; highlights airway involvement
**3**	**Rotsides et al. [5]**	2017	Comparative cohort (multidisciplinary vs	81 children	Pediatric	Not specific to EoE;	Multidisciplinary	Multidisciplinary clinic more effective for recalcitrant	Reflux testing part of aerodiges	Better symptom resolution and

Number	Author	Year	Design	n	Age group	EoE criteria	Laryngeal assessment	Findings	Reflux testing	Outcome
			single specialist)			aerodigestive cohort	airway evaluation	aerodigestive complaints	tive workup	fewer procedures
**4**	**Kim et al. [10]**	2024	Observational transcriptomic study	185 adults	Adults	Confirmed EoE with histology	None	Nonepithelial gene expression correlates with symptom severity	None	Suggests deeper tissue involvement beyond epithelial eosinophilia
**5**	**Chang et al. [13]**	2022	GWAS meta-analysis	2,000+ across cohorts	Mixed	Standard histologic criteria (≥15 eos/hpf)	None	Identifies new EoE susceptibility loci; strengthens genetic architecture	None	Expands genomic risk map for EoE
**6**	**Hironimus et al. [15]**	2025	Pediatric histopathology cohort	64 children	Pediatric	Histologic EoE (≥15 eos/hpf)	None	Mast-cell infiltrates common in active EoE and persist in	None	Suggests mast cells as persistent inflammatory driver

Number	Author	Year	Design	n	Age group	EoE criteria	Laryngeal assessment	Findings	Reflux testing	Outcome
								histologic remission		
**7**	**Gupta et al. [18]**	2025	Multicenter retrospective cohort	347 patients (EGIDs; subset EoE)	Mixed	Histologic criteria for EGIDs/EoE	None	Natural history, treatment patterns, and diagnostic delays in EGIDs	Some patients underwent reflux evaluation	Provides real-world diagnostic/treatment trajectories
**8**	**Fracchia et al. [19]**	2019	Pediatric diagnostic cohort (triple endoscopy)	243 children	Pediatric	Not specific to EoE; chronic cough cohort	Direct laryngeal, bronchial, and esophageal endoscopy	Triple endoscopy improves diagnostic yield for chronic cough	Reflux testing included in workup	Identifies airway/esophageal contributors to chronic cough
**9**	**Sawada et al. [20]**	2023	Case report	1 adult	Adult	Confirmed EoE	Laryngoscopic evaluation	Laryngopharyngeal lesion associated with EoE	None	Demonstrates laryngeal involvement in adult EoE

Number	Author	Year	Design	n	Age group	EoE criteria	Laryngeal assessment	Findings	Reflux testing	Outcome
**10**	**Bae et al. [21]**	2024	Case report	1	Adult	Confirmed EoE	Oropharyngeal exam; swallow assessment	EoE presenting with oropharyngeal dysphagia and aspiration	None reported	Symptom improvement after targeted therapy
**11**	**Trisno et al. [23]**	2025	Pediatric aerodigestive cohort (triple endoscopy)	125 children	Pediatric	Not specific to EoE; recurrent croup cohort	Laryngeal, bronchial, esophageal endoscopy	Esophageal pathology (including EoE) found in recurrent croup workup	Reflux testing included	Triple endoscopy increases diagnostic yield for recurrent croup
**12**	**Bento et al. [24]**	2020	Pediatric case-control study	40 children (approx.)	Pediatric	Histologic EoE	ENT exam; laryngoscopy	Children with EoE show higher rates of ENT manifestations (dysphonia, throat symptoms)	Not consistently reported	Supports ENT involvement in pediatric EoE

Number	Author	Year	Design	n	Age group	EoE criteria	Laryngeal assessment	Findings	Reflux testing	Outcome
**13**	**Huynh et al. [27]**	2025	Pediatric airway-symptom cohort	45 children	Pediatric	Confirmed EoE	Laryngoscopy; airway symptom assessment	Airway symptoms (cough, dysphonia) common in EoE; some improve with EoE therapy	Not systematically reported	Highlights airway involvement and need for ENT evaluation
**14**	**Reddy et al. [28]**	2024	Expert clinical practice update	N/A	Mixed	Includes EoE within immune-mediated esophageal disorders	None	Framework for diagnosing immune-mediated esophageal dysfunction	None	Provides clinical guidance for immune-related esophageal disease
**15**	**Al Abdulqader [33]**	2025	Systematic review	27 studies	Mixed	Standard histologic criteria	None	Reviews emerging noninvasive diagnostic methods (cytosponge, biomarkers,	None	Identifies promising noninvasive diagnostic tools

Number	Author	Year	Design	n	Age group	EoE criteria	Laryngeal assessment	Findings	Reflux testing	Outcome
								saliva, breath tests)		
**16**	**Nguyen HT et al. [34]**	2025	Systematic review + pooled analysis	18 studies	Adults	Histologic EoE	None	Evaluates non-endoscopic biomarkers (ECP, EDN, eotaxin-3, blood markers)	None	Supports biomarker-based monitoring strategies
**17**	**Göldi et al. [37]**	2025	Prospective biomarker cohort	82 adults	Adults	Histologic EoE	None	Fractionated exhaled NO, blood eosinophils, fecal markers correlate with disease activity	None	Demonstrates feasibility of multi-modal noninvasive monitoring
**18**	**El Demellawy et al. [39]**	2022	Pediatric pathology cohort	45 children	Pediatric	Histologic EoE	None	Collins histology score predicts disease severity and outcomes	None	Supports prognostic value of histologic scoring

Number	Author	Year	Design	n	Age group	EoE criteria	Laryngeal assessment	Findings	Reflux testing	Outcome
**19**	**Hernandez et al. [40]**	2020	Imaging review	N/A	Mixed	Standard criteria	Radiologic correlation with endoscopy	Defines CT/MRI features of EoE (wall thickening, strictures) with endoscopic/pathologic correlation	None	Provides imaging-based diagnostic correlations
**20**	**Kubik et al. [41]**	2017	Review / clinical perspective	N/A	Mixed	Standard histologic criteria	ENT exam emphasized	Defines ENT role in EoE evaluation; highlights dysphagia, globus, and cough	None	Encourages multidisciplinary ENT–GI collaboration
**21**	**Rubinstein & Rosen [42]**	2018	Pediatric cohort	89 children	Pediatric	Histologic EoE	Respiratory symptom assessment	Respiratory symptoms (cough, wheeze) common in EoE	Not consistently reported	Supports respiratory manifestations as part of EoE phenotype

Number	Author	Year	Design	n	Age group	EoE criteria	Laryngeal assessment	Findings	Reflux testing	Outcome
**22**	**Kumar et al. [43]**	2019	Review for otolaryngologists	N/A	Mixed	Standard criteria	ENT evaluation discussed	Primer on ENT manifestations: dysphagia, throat pain, and cough	None	Provides ENT-focused diagnostic guidance
**23**	**Moran et al. [44]**	2024	Pediatric aerodigestive cohort (triple endoscopy)	255 children	Pediatric	Histologic EoE	Laryngeal, bronchial, esophageal endoscopy	Factors associated with EoE: atopy, esophageal findings, and airway symptoms	Reflux testing included	Identifies predictors of EoE in aerodigestive population
**24**	**Greifer et al. [45]**	2015	Pediatric aerodigestive cohort	60 children	Pediatric	Not specific to EoE	ENT + airway evaluation	Chronic cough/croup often linked to airway lesions, atopy, and esophagitis	Reflux testing part of workup	Supports multidisciplinary aerodigestive approach

Number	Author	Year	Design	n	Age group	EoE criteria	Laryngeal assessment	Findings	Reflux testing	Outcome
**25**	**Baker et al. [47]**	2023	Pediatric cohort (laryngeal cleft)	34 children	Pediatric	Not focused on EoE	Laryngoscopy; UES manometry	UES dysfunction common after cleft repair; ENT relevance to dysphagia	Some underwent reflux evaluation	Highlights esophageal–airway interplay in dysphagia
**26**	**Cooper et al. [48]**	2012	Pediatric cohort	235 children	Pediatric	Esophagitis including EoE	Airway endoscopy	Atypical croup associated with airway lesions, atopy, and esophagitis	Reflux testing included	Shows esophageal disease contributes to airway symptoms
**27**	**Dellon et al. (KRYPTOS) [49]**	2022	Phase 2/3 randomized trial	~150	Adults & adolescents	Histologic EoE	None	Lirentelimab improves histology; modest symptom improvement	None	Demonstrates anti-Siglec-8 biologic efficacy

Number	Author	Year	Design	n	Age group	EoE criteria	Laryngeal assessment	Findings	Reflux testing	Outcome
**28**	**Rothenberg et al. (MESSINA) [50]**	2024	Randomized controlled trial	211	Adults	Histologic EoE	None	Benralizumab depletes eosinophils; histologic improvement > symptom improvement	None	Confirms IL-5Rα targeting reduces eosinophilia but symptoms lag
**29**	**Collins et al. (LIBERTY EoE TREET) [51]**	2025	Phase 3 randomized trial	~240	Adults & adolescents	Histologic EoE	None	Dupilumab significantly improves histopathologic features (eosinophils, basal zone hyperplasia, spongiosis)	None	Demonstrates robust histologic response to dupilumab
**30**	**Dunn et al. [54]**	2015	Review	N/A	Mixed	Not focused on EoE	Laryngeal exam central	Reviews vocal cord dysfunction (VCD), its triggers, and	None	Provides framework for evaluating VCD, relevant to EoE overlap

Number	Author	Year	Design	n	Age group	EoE criteria	Laryngeal assessment	Findings	Reflux testing	Outcome
								differential diagnosis		
**31**	**Wilkinson et al. [55]**	2021	Clinical review	N/A	Mixed	Not specific to EoE	ENT & swallow assessment discussed	Comprehensive dysphagia evaluation and management strategies	None	Provides diagnostic algorithm for dysphagia (relevant to EoE presentations)
**32**	**Zubair et al. [56]**	2022	Pediatric airway surgery cohort	350+	Pediatric	Not focused on EoE	Airway endoscopy	Trends in pediatric airway surgery; identifies evolving indications	Some underwent reflux evaluation	Highlights airway–esophageal interplay in surgical cases
**33**	**Schoepfer & Safroneeva [62]**	2024	Pharmacologic review	N/A	Mixed	Standard criteria	None	Categorizes drugs as efficacious, likely efficacious, or failed (PPIs,	None	Clarifies therapeutic hierarchy in EoE

Number	Author	Year	Design	n	Age group	EoE criteria	Laryngeal assessment	Findings	Reflux testing	Outcome
								steroids, biologics)		
**34**	**Zeng et al. [64]**	2025	Systematic review & meta-analysis	20+ studies	Mixed	Histologic EoE	None	Biologics (dupilumab, benralizumab, lirentelimab) improve histology; variable symptom response	None	Confirms biologics as effective EoE therapies
**35**	**Kaur et al. [65]**	2023	Pediatric biomarker cohort	92 children	Pediatric	Histologic EoE	None	FeNO correlates with esophageal, gastric, and duodenal eosinophils	Some underwent reflux evaluation	FeNO may serve as noninvasive biomarker for pediatric EoE
**36**	**Górriz-Gil et al. [66]**	2016	ENT-focused clinical review	N/A	Mixed	Standard criteria	ENT exam emphasized	Reviews ENT manifestations: dysphagia,	None	Highlights EoE as relevant to

Number	Author	Year	Design	n	Age group	EoE criteria	Laryngeal assessment	Findings	Reflux testing	Outcome
								globus, cough, throat pain		otolaryngologists
**37**	**Dellon et al. (ACG Guideline) [68]**	2025	Clinical guideline	N/A	Mixed	Histologic EoE	None	Comprehensive adult EoE guidance: diagnosis, therapy, and monitoring	None	Defines U.S. standard of care for EoE
**38**	**Katzka et al. (Cytosponge) [69]**	2017	Two-center diagnostic cohort	86 adults	Adults	Histologic EoE	None	Cytosponge accurately assesses histologic activity; safe and well-tolerated	None	Supports Cytosponge as minimally invasive monitoring tool
